# A conceptual framework for a neurophysiological basis of art therapy for PTSD

**DOI:** 10.3389/fnhum.2024.1351757

**Published:** 2024-04-22

**Authors:** Bani Malhotra, Laura C. Jones, Heather Spooner, Charles Levy, Girija Kaimal, John B. Williamson

**Affiliations:** ^1^Department of Creative Arts Therapies, Drexel University, Philadelphia, PA, United States; ^2^Brain Rehabilitation Research Center, North Florida/South Georgia Veterans Affairs Medical Center, Gainesville, FL, United States; ^3^Department of Clinical and Health Psychology, University of Florida, Gainesville, FL, United States; ^4^Henry M. Jackson Foundation for the Advancement of Military Medicine, Inc. in support of Creative Forces^®^: NEA Military Healing Arts Network, Bethesda, MD, United States; ^5^Center of Arts in Medicine, University of Florida, Gainesville, FL, United States; ^6^Center for OCD, Anxiety and Related Disorders, Department of Psychiatry, University of Florida, Gainesville, FL, United States

**Keywords:** PTSD, post-traumatic stress disorder, art therapy, triple network, therapeutic mechanisms, neurophysiological changes, mechanisms of change

## Abstract

Post-traumatic stress disorder (PTSD) is a heterogeneous condition that affects many civilians and military service members. Lack of engagement, high dropout rate, and variable response to psychotherapy necessitates more compelling and accessible treatment options that are based on sound neuroscientific evidence-informed decision-making. Art therapy incorporates elements proven to be effective in psychotherapy, such as exposure, making it a potentially valuable treatment option. This conceptual paper aims to inform the neurophysiological rationale for the use of art therapy as a therapeutic approach for individuals with PTSD. A narrative synthesis was conducted using literature review of empirical research on the neurophysiological effects of art therapy, with supporting literature on neuroaesthetics and psychotherapies to identify art therapy factors most pertinent for PTSD. Findings were synthesized through a proposed framework based on the triple network model considering the network-based dysfunctions due to PTSD. Art therapy’s active components, such as concretization and metaphor, active art engagement, emotion processing and regulation, perspective taking and reframing, and therapeutic alliance, may improve symptoms of PTSD and prompt adaptive brain functioning. Given the scarcity of rigorous studies on art therapy’s effectiveness and mechanisms of alleviating PTSD symptoms, the suggested framework offers a neurophysiological rationale and a future research agenda to investigate the impact of art therapy as a therapeutic approach for individuals with PTSD.

## 1 Introduction

Post-traumatic stress disorder (PTSD) is a trauma-related disorder that affects up to 6.0% of US civilians and 11.7% of US Veterans (Schein et al., 2021). While psychotherapy is the first line recommended treatment for PTSD and serotonin reuptake inhibitors (SSRIs) are an effective pharmacological approach (Williamson et al., 2021), there is mixed tolerance and response to treatment, necessitating additional treatment options. Exacerbating matters, the presentation of PTSD is heterogeneous, and according to the DSM-5 criteria, there are 636,120 possible symptom combinations that can lead to the diagnosis of PTSD (Cloitre, 2020). Presenting symptoms can include reliving the traumatic event via flashbacks and nightmares, anhedonia, avoidance of situations and reminders of the traumatic event, hyperarousal, fear or anger, and dissociation. There are several empirically supported treatments for PTSD and many patients achieve remission.

Many Veterans with PTSD have a history of traumatic brain injury (TBI), and evidence suggests that this combination may lead to a less robust response to current empirically supported treatment and greater treatment requirements (Wolf et al., 2012; Tanev et al., 2014). This heterogeneity of presentation, including co-morbidities, may require precision treatment methods to optimize efficacy. Approximately 70.4% of people will be exposed to trauma (a criterion A event) in a lifetime, with 4.1% of people going on to develop at least one episode of PTSD (Kessler et al., 2017). Active-duty military members and Veterans are at elevated risk for exposure to traumatic events (Kilpatrick et al., 2013) and TBI, which is a risk factor for prolonged recovery from PTSD (Vanderploeg et al., 2009; Otis et al., 2011; Lange et al., 2020). The mechanistic link between TBI and PTSD is unclear, though it may potentially relate to changes in white matter structures important in regulating emotion and therefore, TBI may result in symptom exacerbation (Williamson et al., 2013; Bottari et al., 2021). Damage to white matter preferentially affects function of frontally connected networks (Sharp et al., 2014; Hayes et al., 2016; Filley and Kelly, 2018) important in emotional regulation, processing speed, attention, and executive functions. Further, white matter plays a key role in regulating limbic system activity, potentially affecting autonomic/hyperarousal features of PTSD. Hypervigilance and physiologic signs of chronic stress (i.e., low heart rate variability, systemic inflammation, and heart disease) are associated with hyperarousal symptoms of PTSD (Kubzansky et al., 2007; Gola et al., 2013; Schneider and Schwerdtfeger, 2020). Hyperarousal symptoms may negatively impact adherence and persistence in psychotherapy for PTSD (Livingston et al., 2020).

Brain networks involved in the expression of symptoms of PTSD, such as hyperarousal include brain stem and associated structures [e.g., connections from brain stem structures to the hypothalamic-pituitary-adrenal (HPA) axis]. Brainstem nuclei, such as the periaqueductal gray, noradrenergic locus coeruleus and dopaminergic ventral tegmental area, play vital roles in facilitating extinction of fear learning through their interconnections with the hippocampus, amygdala, and prefrontal regions, involved in context-specific modulation of fear memories (McNally et al., 2011; Iordanova et al., 2021). Further, vagal efferent and afferent pathways involve critical brainstem structures including the and nucleus of the solitary tract (NTS). The NTS has substantial bidirectional connections to limbic structures and these systems regulate stress response via interaction with multiple neurotransmitter systems (Williamson et al., 2013). Researchers suggest the role of threat-memory encoding at synaptic and circuit levels, and the simultaneous encoding of various aspects of “fear” advocating for non-pharmacological approaches to address consolidation-based impairment of emotional memory and trauma-related memory retrieval to consider reconsolidation interventions (Maddox et al., 2019).

The function of three resting state networks has been identified as potentially important in the expression of cognitive and emotional processes in mental health conditions. Some have called these associations, the triple network theory. While there have been many resting state networks identified, the three with the strongest empirical support in this context are the central executive network (CEN), default mode network (DMN), and the salience/emotion network (SEN) (Akiki et al., 2017, 2018).

### 1.1 Approaches to treatment of PTSD

The mechanisms of psychiatric dysfunction in PTSD have been linked to fear conditioning (Johnson et al., 2012) with dysfunction in extinction processes and safety learning–leading to the reinforcement and preservation of PTSD (VanElzakker et al., 2014). There are several co-morbidities which may prolong and exacerbate recovery including substance use, TBI, and sleep disturbance (Short et al., 2017; Bottari et al., 2023). These co-morbidities can lead to disruptions in memory consolidation, engagement with psychotherapy, and treatment compliance, hindering remission of PTSD (Stickgold and Walker, 2007). PTSD is a learned response that is reinforced behaviorally as a self-preservation mechanism. These survival responses may be protective during active danger. Unfortunately, their persistence is maladaptive in non-threatening contexts. PTSD changes over time and may resolve on its own. However, it is also quite responsive to psychotherapy. Recommended treatments for PTSD include exposure-related psychotherapy and pharmacological intervention (e.g., SSRIs) (Williamson et al., 2021). While psychotherapy has proven to be effective for PTSD (*g* = 1.14; Watts et al., 2013), there are significant rates of dropout (up to 18.28%) for trauma-focused PTSD treatment (Imel et al., 2013). Moreover, many people who complete treatment continue to have clinically significant symptoms of PTSD as well as recurrence. Sleep disruptions often persist even with clinically significant response to psychotherapy (Bottari et al., 2023). Elevated dropout rates are often attributed to the trauma component of most psychotherapies causing distress and discomfort. The American Psychological Association (APA) and Veterans Affairs (VA) recommend trauma-focused cognitive behavioral therapy (CBT-TF) or Prolonged Exposure Therapy (PE) as the gold standard of treatment, with additional psychotherapies indicated as potential options for PTSD treatment such as eye movement desensitization and reprocessing (EMDR) (American Psychological Association, 2020; Norman et al., 2023). Suggested pharmacological interventions are like those recommended for anxiety and depression and include, but are not limited to, SSRIs (i.e., fluoxetine, paroxetine, sertraline) and serotonin and norepinephrine reuptake inhibitors (SNRIs) like venlafaxine. Stigma surrounding mental health diagnoses is prevalent in military populations (Mittal et al., 2013; National Academies of Sciences & Medicine, 2018), and multiple studies have found low evidence-based psychotherapy engagement at 11.4% of Veterans referred (Mott et al., 2014) and higher dropout rates among Veterans with PTSD than randomized control trials (RCT) for PTSD in civilians (Zayfert et al., 2005; Schottenbauer et al., 2008).

### 1.2 Art therapy

Given the lack of engagement with empirically supported psychotherapies, high dropout rates, common incomplete remission of PTSD post-treatment, and heterogeneity of responses to treatment, other interventions are necessary. Art therapy is currently used in the Veterans Health Administrations system of care to treat PTSD. Though empirical support is limited through RCTs, art therapy has been used in a range of military populations with reported benefit (Campbell et al., 2016; Walker et al., 2016, 2017; Levy et al., 2018; Jones et al., 2019; DeLucia and Kennedy, 2021; Schnitzer et al., 2021). Art therapy allows for active creative self-expression that integrates non-verbal communication through visual narratives (Berberian et al., 2019). Service to military populations is at the core of art therapy, which developed from treatments for “battle fatigue” (a term that preceded PTSD), in World War II (Howie, 2017) and since has been a part of integrative and primary psychotherapy for patients with TBI and PTSD. Since trauma may be encoded through visual images, sounds and smells, art therapy may be a modality consistent with sensory encoding of traumatic events. Service members and Veterans identify, externalize, and process their psychological difficulties through art therapy, actively engaging the creative process within a psychotherapeutic relationship facilitated by a credentialed art therapist (Campbell et al., 2016; Walker et al., 2016; Kaimal et al., 2018; Berberian et al., 2019). Military service members and Veterans with PTSD and history of TBI report that art therapy enabled them to gain a sense of integrated identity (Walker et al., 2016), self-regulation (Jones et al., 2018), and access to previously blocked memories and insight (Campbell et al., 2016) suggesting successful fear extinction associated with completing art therapy.

Art therapy has demonstrated positive health outcomes in case studies, observational research, and program evaluation studies with military population with TBI and PTSD (Jones et al., 2018; Kaimal et al., 2018). However, despite decades of use and demonstrated positive impacts on specific outcome measures, including anxiety and depression (Abbing et al., 2018; Dunphy et al., 2019; Kaimal et al., 2019), implementation of art therapy is hindered by the lack of rigorous, well-controlled trials that investigate both effectiveness and neurobiological mechanisms of improvement. This conceptual paper aimed to inform the neurophysiological rationale in the use of art therapy as a therapeutic approach for individuals with PTSD. This is accomplished through a conceptual integration of art therapy change factors, comparison of art therapy change-factors to existing standardized treatments for PTSD, and neurophysiological effects.

## 2 Methods

In proposing a neuroscience-informed art therapy conceptual framework to inform future research agenda for work with Veterans for PTSD, we first conducted a literature search to survey the current status of empirical research in the neurophysiological effects of art therapy. The literature search was guided by the question: “What is the neurophysiological empirical evidence base of art therapy, and how can this inform empirical research with individuals with PTSD?”

To identify research studies with neurophysiological outcomes, major databases (Google Scholar, PsycInfo, Medline) were searched using keywords pertaining to biomarkers, functional near-infrared spectroscopy (fNIRS), functional magnetic resonance imaging (fMRI), magnetic resonance imaging [magnetic resonance imaging (MRI)], electroencephalographic (EEG), blood pressure, heart rate variability (HRV), inflammation, cortisol, imaging, neurotransmitter with art therapy for abstract and title search. Abstracts were reviewed from peer-reviewed publications; and research studies published before December 2022 and in English with neurophysiological measures/outcomes were included. In this step, a total of 19 art therapy publications were identified. These included-

• 5 MRI studies (Walker et al., 2016, 2018; Cucca et al., 2018, 2021; Yu et al., 2021)
• 3 fNIRS studies (Kaimal and Ray, 2017; Yan et al., 2021; Kaimal et al., 2022)
• 4 EEG studies (Belkofer and Konopka, 2008; Belkofer et al., 2014; Kruk et al., 2014; King et al., 2017)
• 2 HRV studies (Haiblum-Itskovitch et al., 2018; Abbing et al., 2019)
• 5 Other biomarker studies (Visnola et al., 2010; Kaimal et al., 2016, 2019; Beerse et al., 2019, 2020).

### 2.1 Narrative synthesis

**FIGURE 1:**
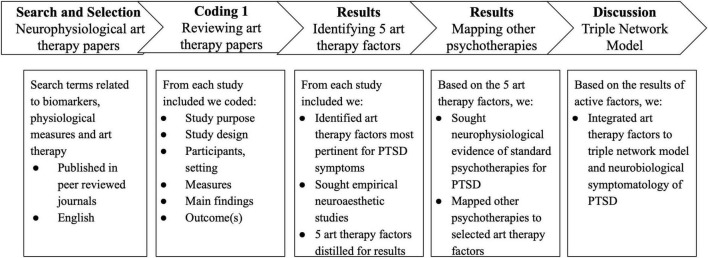
Overview of the steps for conceptual review.

A narrative synthesis method (Edwards and Kaimal, 2016) was used to identify and conceptualize the role of art therapy for PTSD (Figure 1). After search and selection, each art therapy research study was coded for study characteristics identifying study purpose, design, setting, population, intervention, targeted outcomes, and main findings in subgroups of neurophysiological markers such as art therapy and MRI, fNIRS, EEG, HRV, and other biomarkers. Next, the findings on active factors of change for art therapy by de Witte et al. (2021) were considered to identify art therapy processes (common, joint, or specific) such as concretization, symbolism and metaphors, active engagement, artistic pleasure, tactile quality of art, non-verbal expression, modulating time and space, perspective taking, emotion processing and regulation, and therapeutic alliance. We then considered the empirical clusters of therapeutic factors to map on each of the neurophysiological study on art therapy, to find associations in therapeutic factors, neurophysiological effects or outcomes.

To offer a broader perspective on the relationship between therapeutic factors, the team conducted inductive analysis via debriefings and analytical discussions, leading to the identification of five factors relevant to PTSD symptomatology. Considering the small pool of neurophysiological studies in art therapy, findings from empirical neuroaesthetics were reviewed to bolster support in identifying mechanisms and neural bases of visual art engagement based on the five categorized art therapy factors. While these factors are theoretical and potentially interact, they represent a useful framework for developing a structured approach to art therapy. Neuroaesthetics is useful in that this area of work is designed to determine the cognitive and neural basis of art and aesthetic experiences. The known roles of aesthetic engagement in brain function and change may be relevant to mechanisms of art therapy responses in mental health populations. Others have posited these mechanisms in the clarification of intervention strategies and clinical applications of art therapy (King and Parada, 2021). We reviewed studies on creative cognition, and the impact of visual art engagement/production on brain function. We used backward and forward citation searching of the reference lists to supplement supporting evidence. Based on the mechanisms of change in art therapy (de Witte et al., 2021) and the mechanisms reported in empirical studies on art therapy and esthetics, we discuss five art therapy factors and their relevance for treatment of PTSD symptoms. Following this step, the neurophysiological evidence of standard psychotherapies for PTSD as recommended by VA were sought through research papers and user guides for PTSD for psychotherapies to summarize their relation to the five art therapy factors, and to identify similarities/gaps in comparison to art therapy. The selections for creative cognition, neuroaesthetics, and empirical psychotherapy literature were based on their conceptual and analytic association with art therapy factors.

### 2.2 Developing the framework

The principal objective of the conceptual review was to formulate a theoretical foundation research on the function of art therapy. Given the limited content available within the domain of art therapy, supplementary resources from neurophysiological evaluations of psychotherapies and neuroaesthetics research with similar components to art therapy were incorporated to enrich and establish the proposed framework. In the final step, art therapy factors and empirical neuroaesthetics findings were integrated onto the triple network model of PTSD -the altered dynamic between and within the DMN, CEN, and SEN (Akiki et al., 2017). While, there is a broader research base on task dependent change in brain networks (e.g., fronto-limbic networks) in PTSD, the three resting state networks were chosen as they are the most commonly reported fMRI approach found in the literature. This paper provides a conceptual review and outlines future research implications for understanding and investigating the impact of art therapy on PTSD. It achieves this by synthesizing existing literature and proposing ways in which art therapy, informed by neuroscience, could potentially foster resetting adaptive responses to danger and life-threat among individuals with PTSD.

## 3 Active factors in art therapy relevant for PTSD

Art therapy is a form of psychotherapy that uses the creative process, artmaking, and applied psychological theory to address personal and relational treatment goals and enhance mental wellbeing of the individual, their families, and communities (American Art Therapy Association, 2017). Art therapy is a multisensory psychotherapeutic approach that provides kinesthetic, sensory, perceptual, and symbolic opportunities to engage in receptive and expressive communication (American Art Therapy Association, 2017) which likely contributes to psychological and neurophysiological changes (Hass-Cohen et al., 2014). In the following section, findings of five art therapy factors based on mechanisms of change (de Witte et al., 2021) that are conceptualized as most pertinent for PTSD symptoms are presented. Concurrently, the corresponding evidence from other psychotherapies are mapped and summarized as they relate to these five factors.

### 3.1 Concretization, symbolism and metaphor in art therapy

In a scoping review on mechanisms of therapeutic change of creative arts therapies, de Witte et al. (2021) found that the use of symbolism and metaphors, and concrete representation of abstract content into tangible form are unique factors to creative arts therapies. Specifically, art therapy involves expression and processing of thoughts, emotions, and ideas through imagery onto a concrete visual form. These may emerge as representational artwork, as symbols or metaphors where one thing is conceived in terms of another to understand abstract, emotional, or other experiences (Lakoff and Johnson, 2008). The creative process of using metaphors and symbols allows new insights into the self, others, thoughts, feelings, and situations (Kaimal, 2022). Military populations with PTSD and TBI have reported benefit of art therapy to communicate inner experiences or visual self-narratives on artworks-metaphorically or symbolically (Smith, 2016; Walker et al., 2017; Schnitzer et al., 2021).

An empirical neuroaesthetics study indicates that “metaphor production involves brain systems important in executive control, semantic integration, and self-generated thought” (Beaty et al., 2017, p. 163). Studies on creative cognition indicate dynamic interaction between brain regions associated with executive control and spontaneous thought (Mayseless et al., 2015; Bashwiner et al., 2016; Zabelina and Andrews-Hanna, 2016; Beaty et al., 2017). The studies on metaphor production have been focused on linguistic metaphors, and further evidence on connection between artmaking and spontaneous visual metaphor production during art therapy could be important in understanding potential therapeutic impact of these methods.

#### 3.1.1 Concretization, symbolism and metaphor in other psychotherapies

While metaphors are not an explicit component of many psychotherapies, they are often employed for making connections between topics, novel thinking, and emotion processing (Levitt et al., 2000; Stott et al., 2010). Metaphors are used by both the therapist and the patient. Therapists often use verbal metaphors in psychoeducation, homework, and topic conceptualization. Use of metaphor has been heavily studied in cognitive behavior therapy (CBT) (Mathieson et al., 2020; Malkomsen et al., 2021), as well as in acceptance and commitment therapy (ACT) (Stoddard and Afari, 2014), CPT (Laska et al., 2013), dialectical behavior therapy (DBT) (Linehan, 2014), hypnosis (Short et al., 2005; Short, 2018), narrative exposure therapy (Neuner et al., 2020), and psychodynamic therapy (Borbely, 2004). Metaphor has proven powerful enough to warrant emphasized training in CBT (Mathieson et al., 2018).

Relating to concretization, some psychotherapies (i.e., CBT, imagery rehearsal therapy [IRT], cognitive processing therapy with written account [CPT+A], and narrative exposure therapy [NET]) involve creation of tangible products, with the most common “concrete” byproduct a written document (Kushnir and Orkibi, 2021). Interestingly, though the research base is scanty, IRT appears better able to target sleep disruptions in PTSD than other therapeutic approaches (Williamson et al., 2021). Writing enables patients to externalize and monitor their inner thoughts in a concrete form, reinforcing a behavioral pattern of regulating thought process via reasoning through feelings (Beck, 2020). Written externalization facilitates change in perspective and in discovering previously unseen attributes of the stressor. Concrete separation from the stressor in a metaphorical or literal state is intended to help ground clients in the present where they identify and confront their issues instead of engaging in a learned response of avoiding and/or catastrophizing (Moreno, 1987).

### 3.2 Active and creative art engagement: tactile and sensory exploration in art therapy

Actively engaging in artmaking through the choice of varied art media is a specific and unique aspect of art therapy (de Witte et al., 2021). Art therapy facilitates engagement through the physicality of various art materials on a continuum- from structured art media like markers and colored pencils to fluid art media like pastels and paints (expressive therapies continuum, ETC) (Hinz, 2019). Since visual artmaking and imagery are integral parts of art therapy, the visual and perceptual systems of the brain are recruited (King et al., 2019). The tactile and sensory quality of visual art media (Abbing et al., 2018) and the choice of appropriate and specific art materials (Haeyen et al., 2015; Bosgraaf et al., 2020) may contribute to a shift from maladaptive to adaptive responses for clients. This aligns with the framework of adaptive response theory which situates artmaking and the art product as distinct elements in the therapeutic process (Kaimal, 2019).

Art therapy also involves active kinesthetic components that can “induce haptic, proprioceptive, and visual sensations” (Nan et al., 2021). An experimental study examined EEG activity of 14 healthy participants during clay sculpting and drawing recorded over bilateral medial frontal cortex and bilateral medial parietal cortex of each participant (Kruk et al., 2014). Clay sculpting increased gamma power over the right medial parietal lobe and increased theta power over the right frontal lobe. Although a small sample size, these preliminary results suggest impacts of engagement in sculpting on brain regions important in emotional regulation and spatial processing. Other neurophysiological studies on artmaking have also found differences in EEG measurements with significant pre and post drawing differences in the left posterior temporal, parietal, and occipital regions within the alpha band frequency when artists participants used oil pastels (Belkofer et al., 2014). In another study participant’s use of chalk pastels also showed a general increase across all frequencies, with a statistically significant increase in the left hemisphere (King et al., 2017). In patients with Parkinson’s disease (PD) who received twenty sessions of art therapy, there was an improvement in overall visual-cognitive skills and visual exploration strategies as well as general motor function as seen through decreases in visual exploration path length, horizontal fixation variance, and in the number of saccades made during the Benton Visual Recognition Test (Cucca et al., 2021). The participants also showed resting state functional connectivity (fMRI) differences in visual networks (Cucca et al., 2021).

#### 3.2.1 Tactile and sensory exploration in other psychotherapies

Some psychotherapies for PTSD, like EMDR, employ explicit tactile and sensory components. There are two stages of the EMDR protocol: “desensitization” and “resource development and installation (RDI)” (Leeds, 2009). The tactile component of EMDR therapy comes in the form of a bilaterally presented external stimuli that serves as a proposed “focal point” for the client to reference while they reflect on a traumatic experience. The physical stimuli are bilateral, such as tapping oneself on both shoulders at the same time or following the psychotherapist’s hand movements with one’s eyes. The contributions of sensory stimulation, attentional processes, and memory reconsolidation are unclear (Landin-Romero et al., 2018). There is limited support for these elements in the mechanism of action of EMDR. EMDR incorporates exposure, like other empirically supported psychotherapy approaches, and fear extinction learning is a likely mechanism of therapeutic response in EMDR. Physiological evidence for the effectiveness of EMDR comes from Schubert et al. (2016), where they used galvanic skin response (GSR, an indicator of sympathetic nervous system function), heart rate, and respiration measurements on people with PTSD, finding that EMDR resulted in lower GSR and HR responses, suggesting reduced physiological indicators of stress.

### 3.3 Emotion processing and regulation through art therapy

Art therapy involves projection and expression of emotions that can provide therapeutic insights necessary for facilitating treatment goals. In art therapy, the clients see their emotions through the artwork via visual elicitation of past, current, or future states and feelings (Haeyen et al., 2015; Hilbuch et al., 2016; Bosgraaf et al., 2020). Clients in military settings have found art therapy to facilitate trauma recall, increase access to emotions, and enhance representation of self in relation to individual personhood, relationships, community, or society (Campbell et al., 2016; Smith, 2016; Walker et al., 2016, 2017; Lobban and Murphy, 2019).

Art therapists are starting to examine neurobiological mechanisms involved in emotion processing to inform clinical outcomes in art therapy (Czamanski-Cohen et al., 2019). In an ongoing study, Czamanski-Cohen et al. (2020) are examining the mechanisms of art therapy on emotion processing and cholinergic anti-inflammatory processes through a RCT with breast cancer survivors. Haiblum-Itskovitch et al. (2018) examined vagal activity and emotional response to the use of different art materials/mediums. They used the self-assessment manikin visual analog scale (SAM) (Bradley and Lang, 1994) and assessed parasympathetic nervous system (PNS) function via respiratory sinus arrhythmia (RSA). Results indicated an interaction between material and state with a decrease in mean RSA from resting state while painting [F(1, 49) = 26.155, *p* < 0.0005], suggesting significant cognitive, emotional, and physical engagement in the process. The effect size (*n*_p_^2^ = 0.348) indicated that artmaking explained approximately 35% of the variability in parasympathetic reactivity, regardless of art material, indicating changes in emotional regulation processes during the artmaking task. Abbing et al. (2019) assessed anxiety following artmaking with 47 adult women with anxiety disorders with a pre-post RCT (waitlist control group) design using high frequency heart rate variability (HF-HRV, i.e., RSA) via Root Mean Squared Successive Difference (RMSSD) scores. The intervention group showed higher HF-HRV after treatment, consistent with lower anxiety. Art therapy and artmaking may reduce cortisol levels (Visnola et al., 2010; Kaimal et al., 2016; Beerse et al., 2019, 2020) that may improve other psychosocial outcomes of mood, self-efficacy, and perceived stress (Kaimal and Ray, 2017; Beerse et al., 2019). Other preliminary studies have explored blood pressure as a biomarker to examine artmaking as an effective stress reducer (Schrade et al., 2011; Utami et al., 2021). Futterman Collier et al. (2016) found that textile arts have a mood enhancing effect when measured using salivary levels of IL-beta, a proinflammatory cytokine. Overall, there is growing evidence of art making effects on autonomic features and indicators of stress that suggest relevance to positive response in PTSD.

The prefrontal cerebral cortex plays a central role in self-regulation, specifically for reward (e.g., striatum) and emotion (e.g., amygdala) (Kelley et al., 2019). In examining the regulatory effect of drawing on negative emotions, Yan et al. (2021) used autobiographical recall, and randomly assigned 59 participants to one of four conditions- drawing after triggering anger, drawing after triggering sadness, calculating numbers after triggering anger, or calculating numbers after triggering sadness. When drawing regulated sadness, the frontopolar area and left dorsolateral prefrontal cortex (dlPFC) showed significant deactivations on fNIRS, more than its effect on anger. Another fNIRS study gave 26 participants (artists and non-artists) three drawing tasks (coloring, doodling, and free drawing) and noted that all three tasks activated the medial prefrontal cortex in comparison to rest condition irrespective of the skill level, indicating implications for visual art stimulating reward perception (Kaimal et al., 2017). A study on artmaking in virtual reality (VR) (rote tracing and creative self-expression conditions) measured prefrontal cortex (PFC) activation and found that the rote tracing task resulted in higher PFC activity suggesting the potential benefits of creative self-expression in reducing conscious control (Kaimal et al., 2022).

These neurophysiological studies indicate the emerging support for visual artistic practices to facilitate alterations in neural pathways through emotion processing and regulation, and positive emotional experiences that may be a necessary mechanism for recovery for clients with PTSD.

#### 3.3.1 Emotion processing and regulation in other psychotherapies

Although emotional regulation is a frequent target in psychotherapeutic treatment due to heightened focus on survival in patients with PTSD (Ford and Courtois, 2020), the inclusion of intentional, explicit physiological involvement to regulate emotions is not common practice in most psychotherapies. The unpredictable and threatening nature of traumatic events can manifest as and cause negative alterations in self-management skills for dealing with stress and social skills. Techniques such as systematic desensitization through progressive muscle relaxation (Wolpe, 1968), deep breathing (Gilbert, 1999), and more recently heart rate variability biofeedback (HRVB) are intended to aid in emotional regulation in anxiety and PTSD treatments. The engagement of HRVB as well as paced breathing approaches increase parasympathetic activity, and can be thought of as parasympathomimetic treatment approaches. A neurophysiological state with greater parasympathetic engagement may be reflected by better inhibition via prefrontal cortex over limbic activity (e.g., amygdala), manifesting as improved emotional control (Henderson et al., 2004; Gevirtz, 2013). HRV is a measure of vagal tone or condition (Porges, 2007). HRVB can be integrated into any psychotherapy such as acceptance and commitment therapy (ACT) and CBT (Gevirtz, 2020), though data on paired therapeutic approaches are limited. Using resting state fMRI to measure functional changes post prolonged exposure, Fonzo et al. (2017) found increased lateral frontopolar cortex activity and connectivity with the ventral striatum and ventromedial prefrontal cortex. These functional changes were associated with better psychological wellbeing (emotional regulation) and a decrease in hyperarousal symptoms likely due to regulation of negative affect impacting the downstream influence of the frontopolar cortex on the ventromedial prefrontal cortex/ventral striatum, suggesting similar mechanisms of therapeutic efficacy on brain function.

### 3.4 Perspective taking and reframing in art therapy

The concrete representation of internal experiences facilitates perspective-taking and reflection (Deboys et al., 2016; Gabel and Robb, 2017; Abbing et al., 2018; Czamanski-Cohen et al., 2019; Bosgraaf et al., 2020). Art therapy can promote perspective-taking and self-awareness through reflection upon art and via artmaking during sessions (de Witte et al., 2021). Perspective-taking and reframing can occur through clarifying feelings and thoughts; exploring least to most anxiety-provoking situations/feelings/thoughts through art (Rosal, 2022); representing a problematic schema, personal constructs or exploring self-efficacy cognitions (Rosal, 2022) or “mastery images” (Morris, 2014, p. 350); and changing distressing images, symptoms or memories that interfere with functioning (Sarid et al., 2017). There is heterogeneity in the use of art therapy for example, the use of mindfulness-based art therapy is prominent (MBAT) (Rosal, 2022). Indeed, mindfulness-based stress reduction (MBSR) which include relaxation, self-management, exposure, cognitive change, and acceptance as core mechanisms (Baer, 2003) when integrated with art therapy has shown positive outcomes for pain management (Czamanski-Cohen et al., 2014; Fritsche, 2014), for oncology patients (Monti et al., 2006), for individuals with anxiety disorders (Sarid et al., 2017) and severe and persistent mental illness (SPMI) in outpatient treatment settings (Herring, 2014).

As an example of art therapy aiding an individual with PTSD to concretely express distress in art, an active-duty military service member externalized the heightened visual and tactile memories of a flashback personified as a “bloody face in bunker” (BFIB) in a mask and physically contained it in a box to overcome the debilitating intrusive traumatic images (Walker et al., 2016). Studies on art therapy and cognitive flexibility are sparse. However, Walker et al. (2018) who used fMRI to assess thalamic connectivity of Veterans suggested further research using- pre-and post-intervention to examine differences. An MBAT study found significant reductions in anxiety and perceived stress, accompanied by a reduction in salivary cortisol in a group of college students in comparison to the control group (Beerse et al., 2020). Furthermore, empirical esthetics researchers propose testing the hypothesis of aesthetic cognitivism- i.e., how art promotes knowledge and understanding (Christensen et al., 2023). Future mechanism-based intervention studies will benefit from further examination of neural correlates of perspective-taking as it relates to art therapy and PTSD symptoms.

#### 3.4.1 Perspective taking and reframing in other psychotherapies

Other psychotherapies facilitate perspective-taking via externalization which is a form of cognitive reframing that works to challenge negative thought patterns through distancing the patient’s issue from the self. By referring to the issue as a separate entity instead of a characteristic of the patient, it helps the patient develop an internal locus of control. Psychotherapies that employ externalization are CBT, CPT, and NET. In NET, externalization is achieved through restructuring the trauma-survivor life story (or narrative) such that the trauma exists only as an event along the individual’s timeline (Neuner et al., 2020).

Cognitive restructuring, which involves changing emotional experiences via identification of dysfunctional thoughts, is also commonly employed in psychotherapies (Marks et al., 1998; Bryant et al., 2008; Watkins et al., 2018). This can result in less catastrophizing, an increased locus of control, and improved emotional regulation as seen on neurophysiological measures. Improvements in emotion regulation are associated with reductions in autonomic indicators of hyperarousal, for example, increasing resting HF-HRV (Denson et al., 2011).

### 3.5 Therapeutic alliance in art therapy

The profession of art therapy is characterized by active artmaking and the creative process within the psychotherapeutic relationship with an art therapist (American Art Therapy Association, 2017). The positive patient-therapist relationship or therapeutic alliance is a commonly researched factor of therapeutic change (de Witte et al., 2021), with safety of environment and opportunity for emotional release as essential components. Strong therapeutic alliance has been found to be effective and has been a suggested mechanism in art therapy (Bosgraaf et al., 2020; Keidar et al., 2020). Bosgraaf et al. (2020) indicated three patterns of therapist behavior that related to psychosocial outcomes. These were: non-directive therapist behavior where the therapists showed mainly a following-and-facilitating-attitude toward the clients; directive behavior where the therapist showed an active and leading role; and a mix of these two types (eclectic). These three categories were then mapped on to outcomes of internalizing problems, externalizing problems, and social problems in children and adolescents. However, no neurophysiological studies examining therapeutic alliance in art therapy research were identified.

#### 3.5.1 Therapeutic alliance in other psychotherapies

Therapeutic alliance in psychotherapy is based on three tenets of therapist-client relations proposed by Bordin (1979): bond alliance, goal alliance, and task alliance. Anxiety provoking exposures are a frequent component of psychotherapy for PTSD and a well-developed alliance (particularly task alliance) with the therapist was most related to reduced anxiety symptoms and increased engagement (Hagen et al., 2016; Wheaton et al., 2016). Besides enhancing engagement in psychotherapy, higher levels of therapeutic alliance can lead to better treatment adherence and therefore outcomes (Simpson et al., 2011). Hoffart et al. (2013) found that higher levels of bond and task alliance were more predictive than goal alliance of PTSD symptom resolution. The hypothesized importance of therapeutic alliance has been further underscored due to interpersonal dysfunction (Cloitre et al., 1997) which relates to avoidance, mistrust, and negative emotional arousal in PTSD. In studies specific to therapeutic bond using PE as treatment for PTSD, it was found that better alliance at the beginning and throughout treatment was predictive of better resolution of PTSD symptoms (Cloitre et al., 2004; McLaughlin et al., 2014).

Neurophysiological biomarkers of therapeutic alliance include oxytocin measurements (Zilcha-Mano et al., 2018), skin conductance response (SCR) (Marci et al., 2007), EEG, electrocardiogram (ECG), and heart rate (Stratford et al., 2012). Oxytocin is a hormone that acts as a neurotransmitter and is expressed when people bond socially (Carter, 2003; Zilcha-Mano et al., 2020), and could be an indicator of therapeutic alliance. Measurements of oxytocin taken pre, during, and post manualized psychodynamic therapy for depression showed that oxytocin levels significantly increased during psychotherapy sessions corresponding with independently coded instances of the therapist attempting to strengthen the bond (Zilcha-Mano et al., 2018). Stratford et al. (2012) found elevated parietal activation and reduced frontal activation via EEG during reported high therapeutic alliance, suggesting that the patients were engaging in cognitive and emotional insight, though this is a speculative association. Evidence pointing to therapist-patient alliance via SCR showed a significant positive correlation between patient ratings of perceived therapist empathy with time-tagged SCR concordance (Marci et al., 2007).

## 4 Mapping of art therapy associated factors onto the triple network model

**FIGURE 2:**
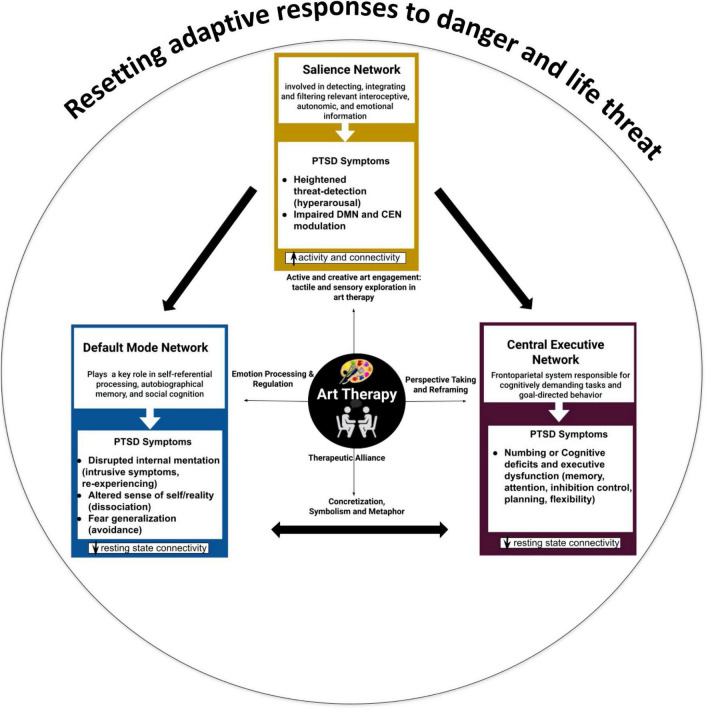
The triple network model in PTSD: neurobiological symptomatology and the role of art therapy active factors in resetting adaptive responses to danger and life threat.

Resting state functional magnetic resonance imaging (rsFMRI) is a common approach to measuring brain function across many populations with several resting state networks identified. Three commonly identified networks associated with cognitive and emotion function are the central executive network (CEN), default mode network (DMN), and salience/emotion network (SEN). There is increasing evidence of altered functioning of these three large-scale brain networks in PTSD and these differences may be associated with specific clinical symptoms (Figure 2; Lanius et al., 2015; Akiki et al., 2017). Acute stress modulates these networks (van Oort et al., 2017) such that activation in the DMN and SEN increases as they work together while the CEN shows minimal activation. In the following section, we summarize and discuss findings from functional and structural neuroimaging studies of PTSD, by primarily focusing on their relevance toward a network-based neurobiological model of PTSD (Akiki et al., 2017). We further discuss the potential of neuroscientifically-informed integration of art therapy factors and neuroaesthetics findings mentioned in the previous section for PTSD interventions. Figure 2 presents this integration that maps the association of art therapy processes on specific brain networks, providing a working framework for the neurophysiological basis of art therapy research in PTSD treatment. It is not intended that researchers focus exclusively on these networks, but given that the relative simplicity in acquisition and established tools for the quantification of these networks, they may serve as a useful indicator of therapeutic success.

### 4.1 Default mode network (DMN)

Default mode network (DMN) is responsible for cognitive processes that occur at rest such as introspection and self-referential thought (Akiki et al., 2017). Structurally, this network is comprised of central areas within the posterior cingulate cortex (PCC), ventromedial prefrontal cortex (vmPFC), and the medial temporal lobe (MTP, including the hippocampus) (Buckner et al., 2008). Decreased connectivity within the DMN is found in numerous psychiatric disorders including PTSD (Bluhm et al., 2009; Daniels et al., 2011; Tursich et al., 2015). It is not known why DMN connectivity is lower in PTSD, however, it is possible that patients with PTSD are less able to relax and this resting state network weakness may reflect this inability. Further, following a Criterion A event there may be disruption of network functions responsible for cognitive reappraisal [lateral orbitofrontal cortex (lOFC) to medial prefrontal cortex (mPFC)] (Akiki et al., 2018) likely leading to symptoms such as negative thoughts and misattribution. According to Mitchell et al. (2005, 2006) and Heatherton et al. (2006), chronic negative thoughts in PTSD are perpetuated by dysfunction in the mPFC which is responsible for assessing personal significance and self-judgement. The mPFC is a component of the DMN and thus people experiencing this symptom may have an associated reduction in DMN connectivity.

Psychotherapies that have longitudinally resulted in increases in DMN and CEN connectivity are CPT (Vuper et al., 2021), PE (Helpman et al., 2016; Fonzo et al., 2017; Zhu et al., 2018), and mindfulness-based exposure therapy (King et al., 2016). Increased CEN and DMN connectivity in the context of hypoactivity from PTSD suggests a therapeutic effect consistent with adaptive change in these systems (Vuper et al., 2021). Thus, these easy to acquire and quantify resting state networks are both related to symptom presentation and are responsive to successful therapeutic intervention.

#### 4.1.1 Implications for art therapy

The lower DMN connectivity demonstrated in the resting state fMRI in Walker et al.’s (2016) study and association of increased DMN connectivity for the injured/traumatic group of service members suggests that art therapy, which has been shown to possibly increase DMN, may potentially balance this system in patients with PTSD. Supporting evidence from neuroaesthetic studies also implies the increased connectivity in DMN for esthetically pleasing experiences (Vessel et al., 2013; Bolwerk et al., 2014; Chatterjee and Vartanian, 2014; Belfi et al., 2019) especially in metaphor production and creative cognition (Ellamil et al., 2012; Beaty et al., 2017).

Studies have implicated DMN in contemplative engagements with artwork when an artwork is viewed as highly moving or resonating (Vessel et al., 2013). Another study demonstrated that training in visual artmaking enhanced functional connectivity in DMN in comparison to the cognitive art evaluation task (Bolwerk et al., 2014). The visual art production group (post-retirement adults) showed greater spatial improvement in functional connectivity of posterior cingulate cortex (PCC/preCUN) to the frontal and parietal cortices which was associated with better psychological resilience measured through a standardized scale (Bolwerk et al., 2014).

Insights into self can be influential when working with people with PTSD with potential issues of the self, other, past, social evaluations and reimagining the future, all of which are implicated in DMN. Treatments that simultaneously target the cognitive and somatic alterations in the self have been suggested to increase DMN connectivity (Lanius et al., 2015). This engagement with somatic and cognitive processes is consistent with proposed understanding of brain networks underlying somatic distress in the context of PTSD (Kearney and Lanius, 2022). Art therapy factors have the potential to address both affective and interpersonal regulation, self-externalization and reprocessing through concretization and metaphorical expression that may be crucial in addressing the identity related disturbances in PTSD. The somatic, emotional, and cognitive nature of art therapy factors warrants further examination to understand neural changes in DMN as a treatment outcome of art therapy for PTSD.

### 4.2 Salience emotional network (SEN)

Another resting state network is the SEN. Hyperarousal symptoms are related to increased activity in SEN at rest. Thus, in PTSD it may be that there is reduced DMN and increased SEN in patients with currently significant symptoms of PTSD. The SEN includes the amygdala, anterior insula (AI), and dorsal anterior cingulate cortex (dACC) (Geng et al., 2016), key structures involved in fear processing and autonomic regulation. Increases in SEN, is associated with lowered threshold of perceived salience leading to inefficient modulation of the DMN-CEN, resulting in network-wide dysfunction between the three networks (Akiki et al., 2017). DMN-SEN coupling dysfunction could lead to greater hyperarousal symptoms (Menon, 2011; Akiki et al., 2017) and is associated with sleep disturbance, elevating risk for intrusive (Dietch et al., 2019) and avoidance (Short et al., 2017) symptoms.

#### 4.2.1 Implications for art therapy

Salience/emotion network (SEN) is crucial in detection of behaviorally relevant stimuli and facilitates dynamic transitions between default and executive systems (Beaty et al., 2017; Vartanian, 2022). The dysregulation of SEN has significant impact on cognition and self-monitoring (Menon, 2011; Lusebrink and Hinz, 2020). In Cucca et al.’s (2021) study on art therapy with patients with Parkinson’s disease (PD) salience network was one of the networks of interest. The functional connectivity values of patients with PD before art therapy were significantly greater than the control group for salience network in the right inferior frontal gyrus (Cucca et al., 2021). While there were no neuroimaging specific results provided for the SEN, neuropsychological test performances on abilities dependent upon the SEN significantly improved. Improvements were notable for decreases in visual exploration path length, horizontal fixation variance, and the frequency of saccadic eye movement during the Benton Visual Recognition Test indicating greater visual orientation efficiency and visuo-spatial skill rehabilitation.

Lusebrink and Hinz (2020) implicate the importance of SEN in selection of “affectively charged images” relevant to the self that integrates internal experiences with cognition. They suggest that emphasizing saliency of image selection through perceptual/affective function of ETC could potentially strengthen the activity of SEN. van Leeuwen et al. (2022) relate this conceptualization in relaying a key role on deciding on whether to engage or disengage from an artwork. The art therapy processes such as active and creative art engagement through sensory and tactile exploration of a range of art media in art therapy may facilitate sensory engagement necessary for restoring interoceptive awareness and salience detection in PTSD (Lanius et al., 2015). Patients engaged in art therapy translate their thoughts and perceptual experiences into an action such as drawing, painting, or sculpting involving interaction between perceptual and motor processes. However more art therapy research is needed to examine the functional connectivity in SEN regions following art therapy for patients with PTSD.

### 4.3 Central executive network (CEN)

The third commonly cited resting state network is the CEN. The CEN allows for cognitive control over emotions and is engaged during effortful cognitive tasks (Menon, 2011). In PTSD, the CEN (anatomically the dlPFC and lateral posterior parietal cortex) shows decreased activity and associated network connectivity which then potentiates disruption of the SEN’s top-down regulation (Seeley et al., 2007; Akiki et al., 2017). The resulting SEN dysfunction leads to a poorly modulated network leading people with PTSD to experience symptoms of hyperarousal and cognitive deficits characterized by executive dysfunction. Indeed, commonly found cognitive deficits of people with PTSD are in selective attention, response inhibition, and memory (Akiki et al., 2017; Dossi et al., 2020).

#### 4.3.1 Implications for art therapy

Impacts of art therapy and artmaking on executive function, visuospatial, and socio-cognitive processing are surfacing (Abbing et al., 2019; Cucca et al., 2021; Yu et al., 2021). Yu et al. (2021) reported a significant increase in cortical thickness via structural MRI scans along with cognitive gains in immediate memory and working memory in older adults with mild cognitive impairment when assessed after 12 weeks of art therapy (*n* = 16). Improved visual-cognitive skills post art therapy were also reported for individuals with PD in preliminary research (Cucca et al., 2021). While functional connectivity studies on effect of art therapy on CEN are limited, Abbing et al. (2019) examined behavioral and cognitive executive functioning with self-report questionnaires and performance-based measures amongst women with anxiety disorders. Their study demonstrated improvements in some aspects of self-reported daily executive functioning (emotion control, working memory, planning/organization and task monitoring). However, no significant differences were noted in cognitive performance of executive functioning; stress responsiveness and down regulation of stress as measured with HRV (Abbing et al., 2019). Exposure to art-related stimuli in art therapy may enhance imagery related encoding strategies transferable to other contexts such as immediate memory and immersive flow experience associated with enhanced focus and sustained engagement. Art therapy, with its emphasis on symbolizing, metaphorizing and cognitive components of perspective taking and reframing, planning, decision making, and problem solving through the artworks may contribute to CEN remediation in PTSD.

The large-scale network dysfunction in PTSD further suggests increased network coupling, or dedifferentiation. For example, a hyperconnected SEN is linked to inefficient DMN-CEN modulation, whereas enhanced CEN to DMN connectivity is associated with treatment response suggesting “an acquired resilience” (Akiki et al., 2017, p. 81). An individual’s engagement in art, its relevance, and meaning are also likely dependent on dynamic and integrated neural activity across the triple networks. There is emerging evidence from neuroaesthetic empirical studies on creative cognition (Beaty et al., 2017) and visual artmaking (Ellamil et al., 2012; De Pisapia et al., 2016) that have demonstrated an increased cooperation between the DMN and CEN using fMRI. Rather than the notion that DMN and CEN typically exhibit an antagonistic relationship, these studies are suggesting that “large-scale networks show dynamic reconfigurations during cognitive processes such as self-regulation, emotion regulation, memory suppression” (Beaty et al., 2017, p. 168). Studying such interdependent interactions between networks in PTSD may be crucial to understanding the relevant mechanisms of the disorder to then inform treatment intervention.

## 5 Resetting adaptive responses to danger and life threat in PTSD

Autonomic dysfunction plays a role in PTSD etiology, symptomology, treatment, and protective factors. Following a criterion A event and maladaptive response, the response of the hypothalamic-pituitary-adrenal axis may become dysregulated fostering an atypical autonomic “startle” response (Heim et al., 2008). This stress reactivity predisposes people to encode stimuli as stressors facilitating the development of PTSD. Neurophysiological state prior to criterion A exposure may increase the likelihood of developing a maladaptive response. For example, in a large cohort of active-duty Marines assesses prior to deployment, lower HRV was associated with development of PTSD after criterion A event exposure (Minassian et al., 2014).

There is emerging evidence on a possible stress regulating effect of art therapy that facilitates relaxation, thereby having a dampening effect on arousal (Abbing et al., 2019) and emotion regulation following specific use of art media (like gouache and oil pastels) (Haiblum-Itskovitch et al., 2018). In conjunction with artistic content associated with the patient’s traumatic experience, fear extinction learning may be facilitated less aversively than in other psychotherapies. Increased resting HF-HRV and reduced sympathetic indicators in response to stress (e.g., electrodermal activity) may indicate improved emotional regulation and could be a useful indicator of art therapy effectiveness on neurophysiological features of PTSD. Art therapy with military personnel with PTSD has shown to support identity integration; externalization of visual and tactile memories; self-expression of emotionally overwhelming experiences onto the artwork; for development of a coherent narrative from the fragmented memories (Campbell et al., 2016; Smith, 2016; Walker et al., 2016, 2017; Jones et al., 2018; Lobban and Murphy, 2019). Improved stress recovery following art therapy could elucidate treatment mechanisms.

Hyperarousal and intrusion symptoms of PTSD culminate in a physiological response leading to dysregulation of the cardiovascular, sleep, and metabolic systems. This autonomic dysfunction can possibly be attributed to chronic parasympathetic nervous system withdrawal (Porges, 2009) with involvement of brainstem structures important for RSA and norepinephrine production. Functional connectivity in amygdalocortical networks responsible for fear response are specifically affected by CBT (Garrett et al., 2019). Working to recognize maladaptive thoughts and physiological responses is part of exposure-based psychotherapies. As exposures occur in a controlled environment with a therapist, extinction learning happens when the traumatic stressor is decoupled from a negative outcome as new and adaptive memories are consolidated. Medication wise, SSRIs such as sertraline and fluoxetine are the most prescribed medications for PTSD (Stein et al., 2006). Another neurochemical process to consider is potential hormonal differences and effects between males and females. Higher levels of dehydroepiandrosterone and testosterone found in males are associated with greater resiliency to stressors (Olff et al., 2007), whereas lower levels of estradiol were associated with higher risk of developing PTSD for women with a history of trauma (Glover et al., 2012). While it is a hormone, oxytocin functions as a neurotransmitter in the brain and is potentially protective against stress reactivity (Koch et al., 2014).

Imagery and exposure have demonstrated modification of key prefronto-limbic regions (Zhu et al., 2018) as well as improvement in indicators of autonomic state (e.g., increased HRV) (Shah et al., 2013) in PTSD. Art therapy combines elements of successful therapies including imagery, exposure, and symbolic communication to facilitate narrative reframing, perspective-taking, reflection, and social engagement that potentially offers unique neurophysiologically relevant advantages over other treatments. However, art therapy’s effectiveness and its neurobiological mechanisms of improvement need to be empirically investigated to test these hypotheses.

Inappropriate threat detection, defensiveness, and inability to perceive safety are also considered critical for individuals with PTSD. Art therapy has been perceived as a safe intervention, providing a psychological safe space and a safe environment for exploration and processing of experiences that are not easily verbalized (de Witte et al., 2021). It is possible that art therapy with its focus on safety through a secure and strong therapeutic alliance aids in overall adaptive responses instead of a defensive one, furthermore facilitating change related to psychophysiological outcomes in individuals with PTSD.

### 5.1 Brain structural alterations

Within the resting state brain networks (DMN, CEN, and SEN), there are structural brain differences seen in individuals with PTSD. It remains unclear why structural differences in the prefrontal cortex and limbic brain volumes are seen in people with PTSD (Bae et al., 2020), but it is possible that these differences are due to persistent stress signaling in the body and or abnormalities in these brain regions predisposing people to develop PTSD. Causality is unclear. Further, white matter structures connecting these regions also show differences in people with PTSD and anxiety disorders, with reductions in white matter integrity demonstrated in the uncinate fasciculus, a key fronto-limbic pathway (Costanzo et al., 2016; Tromp et al., 2019), which also appears to be associated with greater expression of sleep problems in patients with PTSD and mild TBI (Bottari et al., 2021). While reductions in white matter integrity may reflect damage, increases in structural quality measured with DTI have been shown to relate to the learning of new skills. Increases in fractional anisotropy associated with learning have been captured in as little as six weeks or as long as nine months (Scholz et al., 2009; Schlegel et al., 2012). Schlegel et al. (2015) found that training in art skills such as creative cognition, perception, and perception-to-action lead to reorganization of prefrontal white matter. Neuroplastic mechanisms of art therapy and art therapy related cognitive gains have been demonstrated in fMRI study with patients with dementia showing increased cortical thickness in the art therapy group, relative to controls which positively and significantly correlated with enhanced immediate memory (Yu et al., 2021). These preliminary studies provide evidence of potential long term neural structure alteration of prefrontal regions involved in the development and maintenance of PTSD symptomology that need to be researched and documented.

#### 5.1.1 Implications for practice and research

This conceptual review underscores the importance of conducting mechanistic and neurophysiological research in art therapy to understand how and why art therapy works in treatment for PTSD. This framework provides a synthesis of findings for researchers to consider specific outcome variables and processes to study potential mechanisms of the effects of art therapy. This paper provides a meaningful summary to consider a rationale of how art therapy is expected to work with justifications for specific factors as processes of interest in PTSD treatment and research. The empirical clusters of relevant PTSD therapeutic factors discussed in this paper can encourage researchers to consider methodologies and study designs that can address and elaborate the neurobiological effects and outcomes to demonstrate potential neuroplasticity through art therapy in the short and the long term. Further, the administration of art therapy is highly variable. The use of neurophysiological indicators may help develop reliable and valid approaches to the administration of art therapy via optimization of therapeutic choices in the context of art therapy delivery.

The art therapy factors provide a common language and highlight the need develop and employ methodologically rigorous measures that capture the therapeutic factors and their effects (de Witte et al., 2021). Further, researchers can consider intervention components that address, or are based on the domains discussed in the framework; for example, interventions that actively incorporate metaphors, symbols; or address cognitive reframing through art therapy. Clinicians, educators, and researchers may consider change processes with specific art therapy models such as the Expressive Therapies Continuum (Kagin and Lusebrink, 1978) or art therapy relational neuroscience (ATR-N) approaches (Hass-Cohen and Findlay, 2016). Further research is needed to test the associations of art therapy factors and network-based and physiological changes to demonstrate how and to what extent art therapy can indeed foster adaptive responses to threat, proper identification of threat and help patients achieve remission from PTSD.

## 6 Limitations

This conceptual review has several limitations. To date, only one RCT has been conducted on the use of art therapy for PTSD (Campbell et al., 2016). Thus, there is an opportunity to address this void including assessing how individual differences in the expression and severity of PTSD are linked with response to treatment. The neurophysiological studies on art therapy included in this review are diverse and limited for specific physiological measures or biomarkers. More interdisciplinary research collaborations and funding is needed to expand the field of art therapy to systematically study its neurophysiological effects and to optimize its administration. This review is based on synthesis of art therapy and neuroaesthetic studies. Not all studies in this review explicitly measured the art therapy mechanisms making it questionable to consider outcomes (such as emotion regulation or stress) as therapeutic factors. Even though it is worthwhile to gain understanding of how and why art therapy may work, mechanism research is complex- requiring in-depth understanding of related terminologies of mediators, moderators, common, joint, or specific factors (de Witte et al., 2021). The five art therapy factors discussed in this paper likely interact with each other. For example, therapeutic alliance, which is a common factor in psychotherapies may lead to more engagement in sensory and tactile exploration of different art media, which may in turn facilitate perspective taking that may enhance emotion regulation. Clarifying and examining such intervening and interrelated factors calls for methodological sophistication in art therapy mechanism research.

The sample sizes in many studies of neurophysiological impacts of art therapy were small, potentially leading to unreliable conclusions. Clearly, larger sample sizes are desperately needed. Further complicating conclusions, there were few control groups and comparison groups (e.g., comparing psychotherapy, medication, and art therapy). Additionally, the metric upon which studies derived results (such as physiological indices and or questionnaires) was varied leading to difficulties in comparisons across studies. Regarding the design of this conceptual review, the open-ended process through which we found support and potential weak points for applying art therapy in treatment of PTSD allowed for the development of theory not confined to strict search terms.

Moreover, the findings of this paper are based on a conceptual review of art therapy neurophysiological studies. It includes a representative sample of research in other psychotherapies strongly recommended by APA and neuroaesthetics for conceptual mapping in need to support evidence-informed decision-making for art therapy research with PTSD. It is likely that our searches failed to identify studies wherein neurophysiological, or biomarker keywords or terms were not included. Other psychotherapies with emerging evidence such as Brief Eclectic Psychotherapies, psychodynamic therapies, Deep Brain Reorienting (Kearney et al., 2023), and other creative arts therapies like music and dance therapy, have not been underscored in this paper for comparison. Also, while narrative synthesis allowed the inclusion of heterogeneous evidence for the conceptual mapping, it is susceptible to selective reporting (Campbell et al., 2019). The sample of included studies in our review is small, with the potential for bias. Future studies can consider reporting systematic reviews and metanalysis with risk of bias assessments for robust comparisons between studies.

## 7 Conclusion

In this conceptual review, we aimed to inform the neurophysiological rationale in the use of art therapy as a therapeutic approach for individuals with PTSD. This conceptual review underscores the neurophysiological empirical evidence for art therapy through identification of five essential factors as they connect to PTSD symptomatology. Furthermore, the art therapy factors have been mapped onto commonly measured resting state networks with well-established quantification methods to inform network-based dysfunctions due to PTSD and to facilitate easier translation of these findings to larger scale research projects. This provides an underpinning rationale for studies on art therapy for populations with PTSD and for subsequent research to develop effective interventions that address treatment outcomes and help identify the possible mechanisms of change. This review provides an initial framework that integrates empirical art therapy research with neurophysiological processes in PTSD.

## Author contributions

BM: Conceptualization, Data curation, Investigation, Methodology, Visualization, Writing – original draft. LJ: Conceptualization, Data curation, Investigation, Writing – original draft. HS: Writing – review & editing. CL: Writing – review & editing. GK: Conceptualization, Funding acquisition, Supervision, Writing – review & editing. JW: Conceptualization, Funding acquisition, Supervision, Writing – review & editing.

## Author disclaimer

The opinions contained herein represent the private views of the authors and are not to be construed as official or as reflecting the views, opinions, or policies of the Henry M. Jackson Foundation for the Advancement of Military Medicine, Inc., the Department of Defense, the National Endowment for the Arts, or the US Government. Mention of trade names, commercial products, or organizations does not imply endorsement by the US Government. This material was created free of branding or market affiliations. The authors are operating solely as contributors. The work of John Williamson was authored as part of their official duties as an employee of the United States Government and is therefore a work of the United States Government. In accordance with 17 USC. 105, no copyright protection is available for such works under US Law.
